# “They ignore social issues”: understanding the diversity of perspectives on plant gene technologies in Indonesia

**DOI:** 10.1007/s00299-025-03564-0

**Published:** 2025-07-22

**Authors:** Emily A. Buddle, Gloria Fransisca Katharina Lawi, Joan Leach

**Affiliations:** 1 School of Agriculture, Food and Wine, University of Adelaide, Adelaide, SA Australia; 2 ARC Training Centre for Future Crops Development, University of Adelaide, Adelaide, Australia; 3 Research Center for Biotechnology, Universitas Gadjah Mada, Yogyakarta, Indonesia; 4 Centre for the Public Awareness of Science, Australian National University, Canberra, ACT Australia; 5 ARC Training Centre for Future Crops Development, Australian National University, Canberra, Australia

**Keywords:** Gene editing, Biotechnology, Agricultural crops, Socialisation, Indonesia

## Abstract

Genome-editing (GE) technologies are often described as a promising tool for improving agricultural crops, alongside their expanding applications in food and medical research. However, as scientific advancements in GE crops accelerate, there is growing concern that these technologies may follow a similar trajectory to genetically modified organisms (GMOs)—where innovation outpaces public engagement, potentially leading to scepticism and resistance. There are also issues with innovation occurring in domains outside of their target locale, particularly where research and development for uses in the Global South is occurring in the Global North. Thus, there is an important opportunity to conduct better community engagement and technology socialisation in diverse locales. For example, in Indonesia, where food sovereignty is a national priority, understanding public and institutional attitudes toward biotechnology is essential for shaping effective policy and regulation. This paper draws on the first qualitative exploration of Indonesia’s evolving regulatory landscape for gene technologies. Through interviews and media analysis, we examine the perspectives of key stakeholders, including government agencies, NGOs, advocacy groups, and scientists. A recurring theme in our findings is the challenge of “socialisation”—the need for broader public awareness and dialogue about gene technologies, their purpose, and their potential role in Indonesia’s agricultural future. We argue that meaningful engagement must occur early in the development process, within the specific social and cultural contexts of Indonesia, to ensure that biotechnology aligns with local needs and values. By integrating social perspectives into regulatory and research agendas, Indonesia can better navigate the complexities of GE crop adoption and governance.

## Introduction

Many believe that genome- or gene-editing (GE) technologies provide great opportunities for the improvement of agricultural crops, among other applications in food and medical research. Scientists, particularly those who are working on the development and implementation of GE technologies, argue that these techniques are different from those used in genetic modification (GM) (e.g., Jones et al. 2022). Instead, these so-called “New Breeding Technologies” (NBTs) (also referred to as New Genetic Technologies—NGTs) have emerged, based on targeted mutations using tools such as CRISPR-Cas9. More specifically, among crop scientists, there is a great deal of enthusiasm about the potential applications for GE in enhancing crops, with the hope and promise of improving food and nutritional security in developing parts of the world by altering staple crops (Jones et al. 2022; Zaidi et al 2019).

While the scientific community continues to pursue the development of these GE crop varieties with great haste, one cannot help but wonder whether NBTs will become a “GMO 2.0 scenario”, where the science continues to develop without sufficient engagement with other parts of the community to ensure the resulting product is accepted and trusted. While there has been some research to explore community and consumer attitudes towards GE, most studies have been concentrated in the Global North with very little research conducted to understand attitudes toward biotechnology in the Global South (see Strobbe et al. 2023 for an overview). This is particularly problematic when scientific communities within the Global North are leveraging the purported benefits these technologies have for the Global South to attract funding and pursue their science, without considering the unique social and cultural contexts of these countries (International Science Council 2018).

Based on the need for engagement with communities about GE in the Global South, this paper is based on the first qualitative exploration of the complex and evolving regulatory landscape of gene technologies in Indonesia. Using interviews and a desktop-based media analysis, we captured the perspectives of key groups with vested interest in the use of gene technology in Indonesia, including government agencies, NGOs, advocacy groups and scientists. Many of our participants specifically highlighted “the problem of socialisation” whereby there is a need to better understand what role (if any) gene technologies may play in Indonesia. It was apparent that more work is needed to understand if and how biotechnology could be adopted at a grassroots level, particularly due to the dominance of smallholder farmers. It was clear that none of our participants were keen to assume responsibility for greater public engagement, and yet participants were nearly unanimous in suggesting that public engagement and education are critical to the effective socialisation of gene technologies.

While this discussion paper primarily calls on those who work in NBT development in agricultural crops, there is just as much of a need for us in the social sciences, science and technology studies, science communication and related fields to engage with our natural science colleagues to better socialise science, particularly socially and ethically ‘controversial’ science. This engagement is essential to prevent an overemphasis on scientific advancement at the expense of public trust and to ensure expectations of science align with those of the broader community.

## The Indonesian context

Indonesia, a member of the Association of Southeast Asian Nations (ASEAN) and the world’s fourth most populous country, has a diverse agricultural sector shaped by its 17,000 islands and varying climatic zones. Approximately 41 million people work in agriculture, with small-scale farmers constituting around 93% of the farming population (FAO 2018; Universitas Gadjah Mada 2024). The average small family farm is 0.6 hectares, supporting 5–6 household members (FAO 2018). Rice is the main staple food crop, grown in irrigated or lowland systems, where highland areas support cash crops such as coffee, tea, palm oil and vegetables (FAO 2018). Despite increasing food crop production, Indonesia remains a net importer of grains and other agricultural commodities.

Indonesia has a history of both approving and restricting genetically engineered (GE) crops. The first commercial cultivation of Bt cotton occurred in 2001–2002, but Monsanto withdrew in 2003 due to annual regulatory approval requirements and limited growing areas (Amirhusin 2022). Between 2003 and 2021, no commercial GM food crops were grown in Indonesia, except for sugarcane grown in limited areas belonging to the Indonesian Government (Amirhusin 2022). However, corn, potato and sugarcane have recently been approved for commercial cultivation, with the uptake and utilisation of these species set to increase in the medium term (United States of America Department of Commerce 2024).

Despite the limited commercial production of GE crops, Indonesia is a major importer of GE crops, including soya bean and corn for human consumption and livestock feed. In 2022, over 2.3 million metric tonnes of soybeans for human consumption were imported, largely from the United States for the production of tempeh and tofu (Rahayu 2023). The livestock sector also heavily relies on the importation of GM soyabean meal from Argentina and Brazil, and GE corn from the United States (Rahayu 2023). Indonesia is also one of the world’s largest cotton importers, with over 490,000 metric tonnes imported in 2022, with most being Bt cotton, supporting the country’s textile industry (Rahayu 2023).

The large quantities of food and fodder imported into Indonesia have triggered a shift in the country’s policy landscape. Former president, Joko Widodo, publicly supported GE crops, particularly soya beans, to boost domestic production (Rahayu 2023). While newly elected president Prabowo Subianto has yet to make a direct statement on GE crops, his administration prioritises food security. His flagship “free nutritious meals” program, targeting 83 million children, will receive daily nutritional food supported by up to 30% of the Indonesian National State Budget (Cabinet Secretariat of the Republic of Indonesia 2025). At the same time, Indonesia’s food system is evolving. The introduction of “fish milk”—a protein alternative driven by dairy shortages and the need for the free meals program—has sparked debate. While some consumers appreciate the taste, nutrition experts question its ultra-processed nature and sugar content (Lumbanrau Lubis 2024). This bold shift in food innovation may signal increasing openness to GE crops as part of Indonesia’s food security strategy.

There is also a nascent Indonesian research community seeking to do gene technology research in the country to develop crop varieties that is suitable for Indonesian growing conditions. The National Research and Innovation Agency (BRIN) was appointed as the agency responsible for leading research and technology under the Presidential Regulation No. 78/2021, and has recently commenced genome editing in rice, sorghum, wheat, cassava, onion and chilli (Rahayu 2023). Alongside a dynamic international landscape dominated by large corporate agribusiness that have vested interests in supplying seed developed using gene technologies to countries with growing economies, these conditions are seen as favourable for the development and adoption of agricultural biotechnology in Indonesia.

While there is increasing interest and emphasis placed on domestic production of GE crops, public attitudes toward GE technology in Indonesia remain complex. Past studies offer conflicting perspectives: a 2007 survey has indicated that attitudes toward GM by those working in the agricultural science sector were nearly overwhelmingly positive (Februhartanty et al. 2007), while a 2006 study found significant concern amongst the science sectors and farmers in Indonesia (Torres et al. 2006). A more recent survey published in 2020 found varying levels of knowledge, attitudes and trust among eight different public groups: students, scientists, non-government organisations, media, policy makers, consumers, producers and religious scholars (Hidayat et al. 2020). Furthermore, a 2023 USDA report states that public acceptance of gene technologies is high based on a consumer survey conducted in 2020 (USDA 2023). With regards to who is trusted in this domain, a survey conducted in 2020 showed that university scientists were the most trusted source of information, with policymakers, scientists and farmers identified as the stakeholder groups most concerned about agricultural biotechnology (Tome 2021). Furthermore, at the recent National Biotechnology Seminar in Jakarta, the Indonesian ministry officials flagged concern about the wide range of attitudes to gene technologies and a need for ‘socialisation’ of approaches to the technology. However, previous research has relied on survey methods which tell us what potential issues are, but do not illuminate why they are issues, which limits the researcher’s ability to explore the complexity of public attitudes. These findings emphasise the need for deeper, qualitative empirical research to better understand the complexities of attitudes toward the use and regulation of gene technologies in Indonesia.

## Method

This research was approved by the Human Research Ethics Committee at the Australian National Univeristy (H/2023/1448) and  The Ethics Committee on Social Studies and Humanities National Research and Innovation Agency (NRIA) (application number 23082023000003) and conducted in accordance with the Australian National Statement on Ethical Conduct in Human Research (National Health and Medical Research Council et al. 2023). The first stage of this project was a stakeholder mapping exercise, where the authors looked at scholarly literature, recent media publications, policy documents and the regulatory frameworks in Indonesia to identify key discourses on gene technology and the relevant stakeholders with vested interest in the use of biotechnology in agriculture in Indonesia. This step was important to identify which groups should be invited to participate in an interview, and to shape the interview script to ensure that a wide range of perspectives were captured to identify the social and ethical issues relating to biotechnology in agriculture.

The second stage of the research was to conduct a series of interviews with the relevant stakeholders identified in the mapping exercise. Consistent with best practice qualitative research (Denzin and Lincoln 2011), this project used semi-structured interviews to better understand the attitudes of Indonesian actors toward agricultural biotechnology. A total of 11 interviews were held either in person or via teleconferencing software between April and August 2024. Interviews were conducted by Gloria Fransisca Katharina Lawi as a bilingual citizen of Indonesia. Each interview went for between 20 and 80 min, and were either conducted in English or Bahasa, depending on the preference of the participant (a summary of participants is found in Table 1). All audio recordings were transcribed and translated into English (where necessary). Participants were required to be over the age of 18 and have a vested interest in the use of biotechnology in Indonesia, as identified through the media analysis stage of the research. Open-ended questions were posed, allowing for unrestricted responses and facilitating the exploration of the diverse perspectives without the introduction of preconceived ideas (Denzin and Lincoln 2011). The research diverged from traditional grounded theory (Corbin and Strauss 1990), instead adopting a generic inductive qualitative model which combines the data collection process with description, refinement and interpretation of the research questions, alongside purposeful sampling (Hood 2007; Maxwell 2005). All data were treated as rich, narrative text and subject to analytical approaches similar to ‘open coding’ (Maxwell 2005; Thomas 2006). Themes were confirmed with all members of the research team.

**Table 1:** Summary of interview participants

Interview organisation	Role	Overall position on agricultural biotechnology
Indonesia Farmers Union	Advocacy Organisation	Against
Greenpeace Indonesia	Advocacy Organisation	Against
National Farmers and Fisherman Group	Industry Sector	For
Ministry of Agriculture, Republic of Indonesia	Government	For
Centre for Variety Plants and Farming License	Government	For
Biosafety Clearing House/Committee	Research/Regulation	For
Coordinating Ministry of Economic Affairs	Government	For
Indonesian Consumer Organisation	Civil Organisation	Against
Crop Life Indonesia	Industry Association	For
Female Farmer 1	Individual Farmer	For
Female Farmer 2	Individual Farmer	For

## Results: a diversity of views

Unsurprisingly, our stakeholder mapping exercise and subsequent interviews with relevant stakeholders highlight that there is a diversity of views toward the use of agricultural gene technologies in Indonesia, aligning with previous survey results (e.g., Hidayat et al. 2020). Scientists’ views were found to dominate the discussion in the stakeholder mapping exercise, using the media to advocate to the Indonesian government for the need for regulatory reform to allow for greater use of gene editing technologies, furthering the claims that the technology is safe and has an important role in assisting Indonesia with challenges associated with food security and climate change. These views were echoed in the interviews with Indonesian Government representatives, and have been echoed in other locales, particularly with a change in biotechnology methods through the recent development of gene editing reinvigorating debates on how gene technologies should be regulated. Scientists in the field often compare the results of GE as comparable to naturally occurring mutations in living organisms, which do not involve the introduction of genes from other organisms, improving the perceived safety of the resulting food product. For those reasons, scientists generally advocate for a relaxed or the absence of a regulatory framework for GE (Ishii and Araki 2017; Ludlow 2021). Although gene technology regulation in Indonesia has typically used the precautionary principle, the recent change in government may result in a shift in approach toward regulation. This is important to consider, particularly given the role that scientists play on government advisory committees and the potential for minority voices to remain absent from the conversation about appropriate and trusted regulation of gene technologies in Indonesia.

Interestingly, negative sentiment in Indonesia toward gene technologies is not necessarily about the safety of the technology and resulting food, but more about the broader impacts widespread adoption of gene technology will have on social and cultural structures in Indonesia. Civil society perspectives from farmers and NGOs, particularly evident through our interview data, show a concern for other aspects such as food security, and farmer income and welfare. This is particularly evident in the diversity of opinions that exist amongst farmer groups. The *Ketua Kelompok Tani dan Nelayan *(*KTNA*) (National Farmers and Fisherman group) supports the use of gene editing, but are considered by some in Indonesia to be an organisation that represents businessmen that own farms but do not necessarily manage the farms themselves. Conversely, *Serikat Petani Indonesia* (*SPI*) (the Indonesian Farmer’s Union) were against the use of biotechnology, particularly due to scepticism amongst questions about patent ownership of the seed and the individual autonomy of the farmer, and the impact on biodiversity, as demonstrated by the following quote:

It's been a fight for a long time, SPI has been fighting against this, probably since 2001, 2003 since testing for cotton. It's a lot on that research, yes, funded by Monsanto, we as a farmer's organization reject all aspects of why they use biotechnology, first of all, a large population, growing. Second, there is a reason for climate change, so many food crops are disrupted or fail to harvest, so agricultural production is interrupted, even if the government then does import solutions. From there came the solution to all the problems. Of course, that solution also has to consider environmental aspects, whether GMO technology and genome editing will damage the environment? Second, it's related to the sustainability of biodiversity itself. Because of the abundance of seed engineering technology, then local seed will disappear as a result of engineering, then it implies on the definition of local farm seed, and laboratory seed. It's going to run to patents. This is what the Company then holds research. That's what it means to business, and farmers will depend on the seeds. It's the socio-economic factor of biotechnology. Thirdly, the precautionary principle relates to food safety or safety. We don't know if there's been much research. Fourthly, this is what we observe that the seeds of genetic engineering that ultimately require special fertilizer. So one business unit, there's seeds, then fertilizers, then medicines. Then there's the chance of a new pest with these technologies. – Indonesian Farmers Union.

These diverging opinions amongst what may be considered as the same stakeholder group (i.e., farmers) highlights the importance talking to a range of stakeholders, even in one group. While the USDA study found support amongst farmers (USDA 2023), our research found a greater diversity of perspectives that warrant broader consideration.

Amongst the scepticism expressed by some farmer groups, there were also concerns relating to the social-economic inequality introduced by modified crops. This concern was prominent amongst the civil organization for Indonesian consumers, who mentioned the capital bias from the technology companies that insist that farmers implement the modified crops before deeply knowing and fully informed about the characters of the soil and cultures of the farmers communities.

Genetic engineering is very important in Indonesia, without seeing that the Indonesian people, are not a big commercial cotton farmer. But then he forgot all that and intended to apply technology that was not socially friendly. If you've read the research, or not in the researchers' research that one of the transgenic cottons fails because he's not in touch with the local situation…Ignoring the general situation. Communication can persuade and change the habits of humans to the technology product and development. – Indonesian Consumer Organization

While there is an expressed need for greater socialisation of gene technologies, there is already evidence that socialisation of biotechnology amongst farmers has resulted in a greater adoption of modified crops, such as corn, as exemplified by the following quote:

I already feel that being a farmer is more profitable with biotechnology products. Why do I mention biotechnology products? First, the advantage of planting biotechnological corn during the dry season in my area is that usually, due to the lack of water, the trees become barren, but with biotechnological corn seeds, thankfully, the leaves are lush ... Secondly, the biotechnology seeds do not have caterpillar pests. In neighbouring corn fields, there are caterpillars, but in the biotechnology, corn fields, fortunately, there are no caterpillars at all ... I am from biotechnology corn, thank God we have socialised it to the farmers in [Indonesian] district. Finally, this month there are many orders. We understand it like that, so this biotechnology corn is really good. – Female Farmer 2

Our findings echo those from other locales that show concern and rejection of technology is less about the technology itself and more about the impact it will have on broader social and cultural structures (e.g., Busch et al. 2021; Cummings et al. 2023). In the Indonesian context, negative sentiment toward gene technologies were generally associated with concerns about the impact widespread adoption would have on the structure of the industry that is currently dominated smallholder farmers, where current gene technology access and distribution systems are designed to trap farmers, resulting in them being seen as workers for multinational companies, rather than autonomous beings. Meanwhile, the proponents of gene technology believed that negative sentiment was due to a lack of socialisation and communication about gene technologies within the broader community, and with greater education would come greater acceptance and a reduced need for gene technologies to be subject to stringent regulatory processes.

## Why do we need socialisation of science and technology? And who is responsible?

The strong call amongst those interviewed for a plan to “socialise’ GE technologies, their use, their potential benefits and drawbacks is notable. Just as our interviews suggested a broader diversity of views (and of reasons for these views) that earlier studies, our interviewees seem to suspect there is a lot of work to be done with the publics in Indonesia to understand what the introduction of GE means for their food system. What does this mean, according to our interviewees?

First, there is the tendency from expert groups to assume that if others knew more about the technology itself, concerns and questions would disappear: “Well indeed, we need to do socialization and education to the public as well”—Coordinating Ministry of Economic Affairs. And yet, our interviews found that questions farmers had about the introduction of GE were not predominantly about the technology itself, but rather what it means for the agriculture and food system. So, in this case, ‘socialisation’ must mean a broader dialogue between, for example, experts and farmers about what the introduction of GE means for the farming practices and the types, quantity and quality of food they can produce. In short, it may not be the GE technology that is in question but the alignment of that technology with broader social values in farmers and consumers.

Second, Indonesia is a large and richly diverse nation, and this extends to its agricultural and food systems. While there is much focus on GE as a set of technologies, people do not eat technology, they eat food. As evidenced by the above discussion of ‘fish milk’, some Indonesians may embrace food innovations. There is also evidence that some Indonesians may feel comfortable with the use of GE to produce food as associated with the continued importation of tempeh and tofu, both of which are GM products. However, ‘socialising’ to our interviewees means discussing innovations and GE outside of the ‘bubble’, broadening conversations beyond those who are already proponents of the technology, engaging with communities that are more difficult to ask. In our data, this arose around the distinctive role that women play in both the agricultural and food system—as farmers, as providers, as cooks, as those who make decisions for their families. Socialisation, then, is not a survey or a one-time only conversation, it is an ongoing engagement with groups, like women, who may have shifting views and questions about GE.

Third, our interviewees recognised that ‘socialisiation’ is not a panacea and does not stand in for benefit sharing. Indonesia, like nations everywhere, is struggling with how to resource its innovation system, thus ideas about a GE tax. Our interviewees have specific concerns about benefits flowing offshore and about Indonesians missing out on the benefits that GE might provide. What socialisation means in this context, then, is an appraisal of the costs, risks and benefits to various communities. While historically Indonesia has pursued a precautionary approach, that may be shifting. For our interviewees, innovation was seen to bring possible benefits and researchers particularly were enthusiastic about what those benefits might be. At least one farmer’s group was not enthusiastic as they saw benefits potentially flowing offshore and people like them missing out, and consumer advocates were concerned that the small holder farmer will remain disadvantaged by the barriers to access to GE seed and the potential threat to their livelihood and existence. Socialisation, then, is also about understanding benefit sharing.

Finally, coming to these debates in Indonesia with an Australian-Indonesian lens has been a challenge to the frameworks that have been applied elsewhere. Our interviewees didn’t reach for ‘responsible innovation’ or express worries about ‘engagement’. Rather, they used their own formulation of ‘socialisation’ to draw attention to the rather daunting task of taking GE discussions out of the labs and government regulations to talk about it with a broad audience of Indonesians. And here there is also opportunity for exploring new pathways to innovation as well. While it is a long way from labs in Yogyakarta to farms is Kasepuhan Gelar Alam, there is much to be gained from mutual understanding from GE research scientists and small-hold farmers. Our interviewees explicitly referenced a ‘social’ process for this work, not a technical one. From that, there is optimism that socialisation might be the first step on a path to co-design new solutions for Indonesia’s food system challenges.

## Conclusion and recommendations

Our findings highlight the need for greater socialisation of gene technologies within the Global South through meaningful public engagement and open dialogue beyond the already engaged community, extending engagement with hard-to-reach groups such as women or people residing in rural locales. Scientific advancements do not exist in a vacuum—each country has its own social, cultural, and political context that must be considered to ensure new technologies are accepted and beneficial.

In Indonesia, a key challenge is aligning stakeholder interests around genetic engineering. Effective communication requires scientists to engage diverse audiences—first by addressing public concerns about how agricultural biotechnology will impact their lives and communities, and then by ensuring journalists and media platforms are equipped to convey accurate, accessible information. Another critical issue is ensuring the voices of marginalized communities are heard. For example, female farmers in Indonesia, often overlooked in policy discussions, could be key allies in promoting science and technology due to their deep commitment to community well-being. Scientists must also be open to adjusting their research and application of the technology, working with broader community groups to understand what use of the technology is going to be accepted, rather than assuming all applications will be of use or considered relevant to all. For example, a drought-tolerant crop may be more readily accepted than a crop that has been modified to alter the nutritional quality of the grain.

Encouragingly, scientists in our research recognise the importance of socialisation but often lack the resources or expertise to engage effectively. The current focus on next-generation biotechnologies (NBTs) presents an opportunity to bridge this gap—bringing farmers, consumers, and researchers into the conversation to explore what role, if any, biotechnology should play in shaping the future of food and agriculture in different national contexts.

Ultimately, evidence-based policy must extend beyond scientific research to consider broader socioeconomic, cultural, and political realities. Conversations about agricultural biotechnology need to account for both national priorities and global challenges, and integrate broader perspectives than groups with vested interests. Future research should focus on greater engagement with key stakeholders to gain a deeper understanding of social issues relating to agricultural biotechnology in the Global South and explore ways to enhance socialisation.

## Data Availability

Data will be made available upon request.
